# Recruiting Older Adults With Cognitive Impairments for a Smartphone Intervention During COVID-19

**DOI:** 10.1093/geroni/igab046.124

**Published:** 2021-12-17

**Authors:** Stacey Schepens Niemiec, Lon Schneider, Jeanine Blanchard, Rafael Wagas, Liberty Teodoro, Karen Dagerman

**Affiliations:** 1 University of Southern California, Los Angeles, California, United States; 2 University of Southern California, los angeles, California, United States

## Abstract

The Coronavirus disease 2019 (COVID-19) pandemic has introduced numerous challenges to the global scientific community and has been particularly disruptive to the conduct of ongoing clinical trials. Gerontological studies that focus on older adults with cognitive impairments have endured additional challenges ranging from increased vulnerability of this group to COVID-19, thereby prohibiting study participation, to difficulties in participant engagement as a product of a worsening Digital Divide. The purpose of this talk is to describe the pandemic-related factors that have influenced recruitment and enrollment of older adults with mild cognitive impairment and mild dementia in an ongoing feasibility study of a physical activity smartphone app. We discuss the changes we made to recruitment procedures and the impact those changes have had on the success of enrolling individuals in the study. We conclude with a discussion of feasible strategies and procedural alterations moving forward that may facilitate achievement of enrollment goals.

